# Development and clinical utility analysis of a prostate zonal segmentation model on T2-weighted imaging: a multicenter study

**DOI:** 10.1186/s13244-023-01394-w

**Published:** 2023-03-16

**Authors:** Lili Xu, Gumuyang Zhang, Daming Zhang, Jiahui Zhang, Xiaoxiao Zhang, Xin Bai, Li Chen, Qianyu Peng, Ru Jin, Li Mao, Xiuli Li, Zhengyu Jin, Hao Sun

**Affiliations:** 1 Department of Radiology, State Key Laboratory of Complex Severe and Rare Disease, Peking Union Medical College Hospital, Peking Union Medical College, Chinese Academy of Medical Sciences, Shuaifuyuan No.1, Wangfujing Street, Dongcheng District, Beijing, 100730 China; 2 National Center for Quality Control of Radiology, Beijing, China; 3 AI Lab, Deepwise Healthcare, Beijing, China

**Keywords:** Prostate, Magnetic resonance imaging, Deep learning, Segmentation, Multicenter study

## Abstract

**Objectives:**

To automatically segment prostate central gland (CG) and peripheral zone (PZ) on T2-weighted imaging using deep learning and assess the model’s clinical utility by comparing it with a radiologist annotation and analyzing relevant influencing factors, especially the prostate zonal volume.

**Methods:**

A 3D U-Net-based model was trained with 223 patients from one institution and tested using one internal testing group (n = 93) and two external testing datasets, including one public dataset (ETD_pub_, n = 141) and one private dataset from two centers (ETD_pri_, n = 59). The Dice similarity coefficients (DSCs), 95th Hausdorff distance (95HD), and average boundary distance (ABD) were calculated to evaluate the model’s performance and further compared with a junior radiologist’s performance in ETD_pub_. To investigate factors influencing the model performance, patients’ clinical characteristics, prostate morphology, and image parameters in ETD_pri_ were collected and analyzed using beta regression.

**Results:**

The DSCs in the internal testing group, ETD_pub_, and ETD_pri_ were 0.909, 0.889, and 0.869 for CG, and 0.844, 0.755, and 0.764 for PZ, respectively. The mean 95HD and ABD were less than 7.0 and 1.3 for both zones. The U-Net model outperformed the junior radiologist, having a higher DSC (0.769 vs. 0.706) and higher intraclass correlation coefficient for volume estimation in PZ (0.836 vs. 0.668). CG volume and Magnetic Resonance (MR) vendor were significant influencing factors for CG and PZ segmentation.

**Conclusions:**

The 3D U-Net model showed good performance for CG and PZ auto-segmentation in all the testing groups and outperformed the junior radiologist for PZ segmentation. The model performance was susceptible to prostate morphology and MR scanner parameters.

**Supplementary Information:**

The online version contains supplementary material available at 10.1186/s13244-023-01394-w.

## Key points

- The U-Net model showed a good performance for prostate zonal segmentation.
- The U-Net model outperformed the junior radiologist in peripheral zone segmentation.
- Prostate morphology and MR scanner parameters may affect the model’s performance.

**Supplementary Information:**

The online version contains supplementary material available at 10.1186/s13244-023-01394-w.

## Introduction

Accurate prostate segmentation on Magnetic Resonance (MR) images plays an essential role in many clinical applications related to prostatic diseases. Prostate whole-gland segmentation is frequently performed in MR–ultrasound fusion biopsy, radiotherapy planning, and treatment response monitoring [1–4]. Additionally, prostate zonal segmentation, which refers to the separate delineation of the peripheral zone (PZ) and central gland (CG), is crucial in clinical settings. Zonal segmentation is important for the localization of prostate cancer and surgical planning [3], as well as for standardizing the calculation of prostate-specific antigen density [5]. Furthermore, prostate zonal volume calculation enhances the understanding of urinary obstructive symptoms [6].

Traditionally, the prostate zonal segmentation is performed manually by radiologists on T2-weighted images (T2WI). Nevertheless, manual segmentation is a time-consuming process with considerable interobserver variability [7]. Many researchers have proposed automated segmentation methods for prostate zonal delineation on T2WI using deep learning convolutional neural networks (CNN), which yielded good performance with substantially less time consumption. The Dice similarity coefficients (DSCs) of previously reported models were 0.765–0.938 for CG and 0.640–0.868 for PZ [8–11]. Despite their well-demonstrated feasibility, the applicability of CNN models in external testing datasets has been less investigated, especially the application performance in patients with more advanced prostate cancer. In addition, the factors influencing CNN models’ performance have not been thoroughly analyzed [12]. Therefore, we think it is necessary to validate CNN models’ clinical utility in different external datasets and thoroughly investigate how patients’ clinicopathological characteristics, prostate morphology, and image parameters influence segmentation performance.

In this study, we aimed to develop a 3D U-Net-based segmentation model for accurate and efficient auto-delineation of the prostate PZ and CG on T2WI, and to assess its clinical utility in various external datasets by comparison with a junior radiologist and by investigating relevant factors influencing the model performance, especially the prostate zonal volume.

## Materials and methods

### Datasets

Treatment-naive patients who had undergone multiparametric prostate MRI and subsequent biopsy at our institution between November 2014 and December 2018 were retrospectively enrolled. Patients were excluded if the image quality was poor with artifacts that limited the differentiation between CG and PZ, or if normal prostate margins were difficult to identify due to extensive tumor invasion. A total of 316 patients were finally included. These patients were divided into a training-validation group (n = 178 in the training group, and n = 45 in the validation group, respectively, in each fold of cross-validation) and an internal testing group (n = 93) based on their examination time of MRI.

**Fig. 1:**
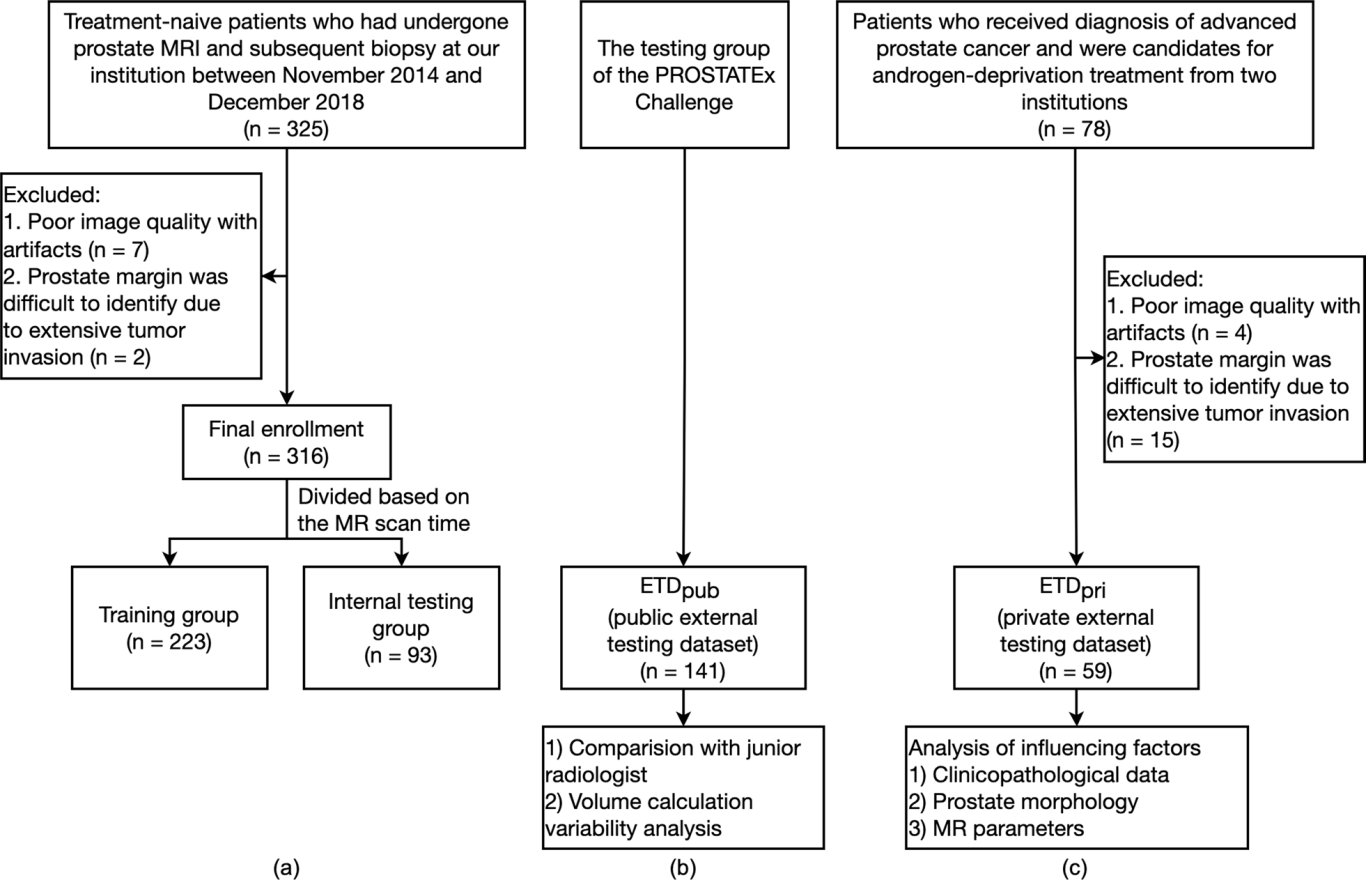
Flowchart of the datasets selection process for the (**a**) training and internal testing groups, (**b**) public external testing dataset (ETD_pub_), and (**c**) private external testing dataset (ETD_pri_)

To fully verify the performance of this model in different patient cohorts, two external testing groups, one public and one private, were employed in this study. The public external testing dataset (ETD_pub_, n = 141) used the testing group of the PROSTATEx Challenge available from *The Cancer Imaging Archive* [13, 14]. The private external testing dataset (ETD_pri_) collected patients from two different centers with various vendors. These patients shared distinct clinicopathological characteristics with the patients in the training group. They received diagnosis of advanced prostate cancer and were candidates for androgen-deprivation treatment. After excluding patients following the same criteria mentioned above, the final dataset included 59 patients. Figure 1 shows the datasets selection process of this study. The institutional review board of our institution approved this retrospective study and waived the need for informed consent.

### Prostate MRI protocol

For patients from our institution, a 3.0T MR scanner (Discovery 750, GE Healthcare) was used. For patients from the PROSTATEx dataset, two different types of Siemens 3.0T MR scanners, the MAGNETOM Trio and Skyra, were used. The MR images in ETD_pri_ were acquired from eight MR scanners with different magnetic field strength (1.5T and 3.0T) of three vendors, including Siemens, GE, and Philips. The detailed MRI acquisition parameters of each group are presented in Additional file 1: Table S1.

### Ground truth segmentation

The patients’ axial T2WI images were collected and manually segmented by an expert radiologist (> 1000 prostate MRI interpreted) to serve as the ground truth; another expert radiologist (> 3000 prostate MRI interpreted) reviewed the images and the segmentations and modified the contour if necessary. Manual segmentation of MR images was performed on the Deepwise Research Platform (Deepwise Healthcare, http://label.deepwise.com). We provide the detailed ground truth segmentation method in Additional file 1: S-1.

### Prostate zonal segmentation model

**Fig. 2:**
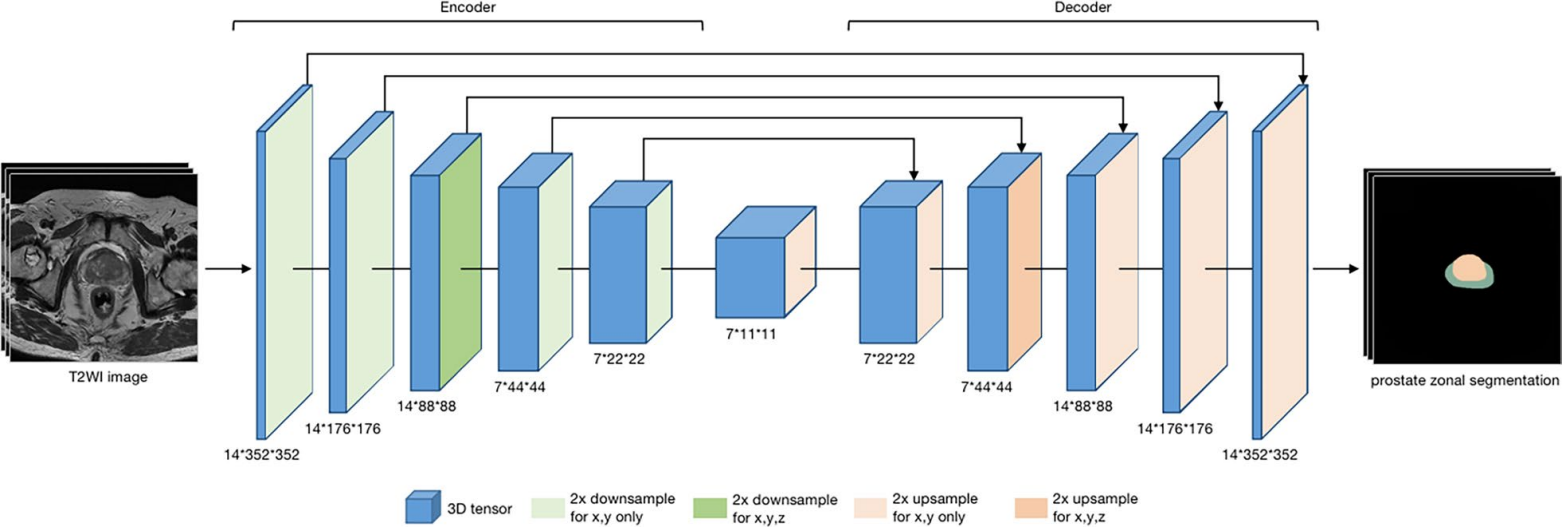
The structure of the prostate zonal segmentation model. The 3D U-Net model was constructed of an encoder and a decoder, and the hyper-parameters of the model were generated from the nnU-Net framework

First, the images were resampled and cropped into the input patch (14 × 352 × 352), followed by an image normalization using z-score. The 3D U-Net–based prostate zonal segmentation model was trained by self-configuring nnU-Net framework [15]. For each pixel of the input image, the model predicted three probabilities, for the non-prostatic region, the CG region, and the PZ region. The structure of the zonal segmentation model is shown in Fig. 2. With an epoch number of 500, an initial learning rate of 0.01, and a batch size of 2, the prostate zonal segmentation model was trained in a five-fold cross-validation procedure. The development process of the zonal segmentation model is provided Additional file 1: S-2.

### Comparison with junior radiologist

To demonstrate the U-Net model’s clinical utility, the model was compared with a junior radiologist's annotation for prostate zonal segmentation. Firstly, fifty cases were selected from the ETD_pub_ using simple random sampling to perform the comparison. Then, the junior radiologist (who had interpreted approximately 100 prostate MRI) manually segmented the CG and PZ on these patients. Taking the expert’s manual segmentation as the ground truth, the performance of the junior radiologist and of the automatic segmentation model among the selected cases were calculated and compared. The prostate volume calculation variability of the junior radiologist and U-Net was also calculated, and the two were compared.

### Analysis of factors influencing auto-segmentation model

To analyze factors influencing the model’s auto-segmentation performance in the external testing dataset, we collected the clinicopathological data, prostate morphology features, and MR acquisition parameters of patients in the ETD_pri_. The clinicopathological data included T stage (T3–4 vs. T2), Prostate Imaging-Reporting and Data System (PI-RADS) score, tumor location (involving both PZ and CG vs. involving one anatomy zone), and lesion maximum diameter. The prostate morphology features included CG volume (CGv), PZ volume (PZv), CGv/whole gland volume (CGv/WGv), WG sphericity, and WG presumed circle area ratio [6]. MR acquisition parameters included MR field strength (3.0T vs. 1.5T), vendor (GE vs. non-GE), slice thickness (> 3 mm vs. ≤ 3 mm), and pixel spacing (> 0.51 vs. ≤ 0.51).

### Statistical analysis

The patients’ characteristics and the metrics’ distributions were described by the median [interquartile range (IQR)] or mean and standard deviation (SD) for quantitative characteristics, and by the absolute and relative frequencies for qualitative characteristics. We calculated the DSC, 95th Hausdorff distance (95HD), and average boundary distance (ABD) to evaluate the performance of our 3D U-Net model. The DSC is widely used to quantify the spatial overlap between segmentations [16], and 95HD and ABD are commonly used to evaluate the boundary errors of segmentation. For the comparison of the model and the junior radiologist annotation, taking the expert radiologist’s segmentation as a reference, the DSC, 95HD, and ABD of the junior radiologist vs. ground truth and the model vs. ground truth were calculated respectively, and compared using a paired-sample *t*-test. The volume variability between U-Net and ground truth, and between junior radiologist and ground truth, was calculated using the intraclass correlation coefficient (ICC) and displayed using Bland–Altman plots. A multivariate beta regression analysis was used to model the DSC for CG and PZ segmentation according to various influencing factors [17]. The analyses were performed using R version 4.2.0 (The R Foundation). The beta regression was implemented using the R package betareg [18]. *p* values lower than 0.05 were considered statistically significant.

The major components of our code are available in open-source repositories or libraries, including nnUNet (https://github.com/MIC-DKFZ/nnUNet) and PyTorch version 1.6.0 (https://pytorch.org/). The manual segmentation masks of PZ and CG in the ETD_pub_ are available on GitHub (https://github.com/LiliXu2022/PROSTATEx_testing_masks).

## Results

### Patients’ demographic characteristics

The mean age in the training group, internal testing group, and ETD_pri_ was 65 ± 8 years, 66 ± 8 years, and 69 ± 8 years, respectively, with median prostate-specific antigen level of 9.4 [IQR 6.3–17.2] ng/mL, 8.9 [IQR 6.1–15.5] ng/mL, and 81.0 [IQR 14.4–207.8] ng/mL, respectively. In the training group, 71.7% of patients had suspicious lesions with PI-RADS score ≥ 3, and 46.6% of patients were diagnosed as prostate cancer (PCa), with most suspicious lesions in the CG. In contrast, the ETD_pri_ contained a higher proportion of PI-RADS 5 lesions (72.9%), with most lesions involving both CG and PZ. The clinicopathological data for the training group, internal testing group, and ETD_pri_ are summarized in Table 1. Because most of the demographic characteristics of patients in the ETD_pub_ were not provided by the PROSTATEx Challenge, their information was not shown in Table 1.

**Table 1:** Clinicopathological characteristics of patients in the training, internal testing dataset and external testing dataset

Variable	Training group (n = 223)	Internal testing group (n = 93)	ETD_pri_ (n = 59)
Age (years)^a^	65 ± 8	66 ± 8	69 ± 8
Prostate-specific antigen (ng/mL)^b^	9.4 [6.3–17.2]	8.9 [6.1–15.5]	81.0 [14.4–207.8]
Prostate volume (cm^3^)^b,c^	49.1 [34.0–70.3]	41.6 [28.3–59.2]	45.2 [32.8–67.3]
*PI-RADS*^*d*^			
1–2	28.3 (63/223)	16.1 (15/93)	0 (0/59)
3	9.0 (20/223)	9.7 (9/93)	11.9 (7/59)
4	29.2 (65/223)	50.5 (47/93)	15.3 (9/59)
5	33.6 (75/223)	23.7 (22/93)	72.9 (43/59)
Non-prostate cancer^d^	53.4 (119/223)	30.1 (28/93)	0.0 (0/59)
Prostate cancer^d^	46.6 (104/223)	69.9 (65/93)	100.0 (59/59)
*Index lesion location (PI-RADS ≥ 3)*^*d*^			
PZ	30.6 (49/160)	28.2 (22/78)	33.9 (20/59)
CG	51.9 (83/160)	43.6 (34/78)	8.5 (5/59)
Both	17.5 (28/160)	28.2 (22/78)	57.6 (34/59)
*T stage*			
T2	–	–	10.2 (6/59)
T3	–	–	39.0 (23/59)
T4	–	–	50.8 (30/59)
Maximum lesion diameter (cm)^a^	–	–	2.8 ± 1.4

^a^Mean ± standard deviation. ^b^Median [interquartile range]. ^c^Volume was calculated from manual segmentation results. ^d^Percentage [%] (absolute value)

*ETD*_*pri*_ Private external testing dataset, *PI-RADS* Prostate Imaging-Reporting and Data System, *PZ* Peripheral zone, *CG* Central gland

### Automated zonal segmentation performance evaluation

The mean DSCs of U-Net for CG in the internal testing group, ETD_pub_, and ETD_pri_ were 0.909 ± 0.044, 0.889 ± 0.064, and 0.869 ± 0.066, respectively, while the DSCs for PZ were lower with values of 0.844 ± 0.095, 0.755 ± 0.092, and 0.764 ± 0.147, respectively. As shown in Table 2, the mean 95HD and ABD were the lowest in the internal testing group, with values of 3.177 mm and 0.575 mm for CG, respectively, and 3.636 mm and 0.555 mm for PZ, respectively, and were the highest in the ETD_pri_ with values of 6.973 mm and 1.224 mm for CG, respectively, and 6.973 mm and 1.300 mm for PZ, respectively.

**Table 2:** Dice similarity coefficient, 95th Hausdorff distance, and average boundary distance of 3D U-Net model on T2-weighted images (mean ± standard deviation)

Structure	CG						PZ					
Datasets	DSC				95HD (mm)	ABD (mm)	DSC				95HD (mm)	ABD (mm)
	Whole zone	Apex	Midgland	Base			Whole zone	Apex	Midgland	Base		
Internal testing group	0.909 ± 0.044	0.856 ± 0.087	0.941 ± 0.033	0.901 ± 0.055	3.177 ± 1.395	0.575 ± 0307	0.844 ± 0.095	0.788 ± 0.133	0.896 ± 0.054	0.832 ± 0.092	3.636 ± 2.701	0.555 ± 0.525
ETD_pub_	0.889 ± 0.064	0.818 ± 0.094	0.937 ± 0.038	0.854 ± 0.118	4.271 ± 2.664	0.963 ± 0.611	0.755 ± 0.092	0.625 ± 0.169	0.818 ± 0.083	0.739 ± 0.140	3.599 ± 3.848	0.991 ± 0.627
ETD_pri_	0.869 ± 0.066	0.811 ± 0.155	0.916 ± 0.056	0.847 ± 0.075	6.973 ± 8.544	1.224 ± 1.485	0.764 ± 0.147	0.651 ± 0.251	0.818 ± 0.202	0.753 ± 0.152	6.973 ± 6.904	1.300 ± 1.442

*ETD*_*pub*_ Public external testing dataset, *ETD*_*pri*_ Private external testing dataset, *PZ* Peripheral zone, *CG* Central gland, *DSC* Dice similarity coefficient, *95HD* 95th Hausdorff distance, *ABD* Average boundary distance

The segmentation performance of the U-Net in different parts of the prostate is also summarized in Table 2. For both PZ and CG, the U-Net showed better performance in the midgland of prostate than in the base and the apex among the three testing groups. In the internal testing group, ETD_pri_, and ETD_pub_, the mean DSCs in the midgland, base and apex of prostate were 0.916–0.941, 0.847–0.901, and 0.811–0.856 for CG, and were 0.818–0.896, 0.739–0.832, and 0.625–0.788 for PZ, respectively.

### Comparison with junior radiologist

**Fig. 3:**
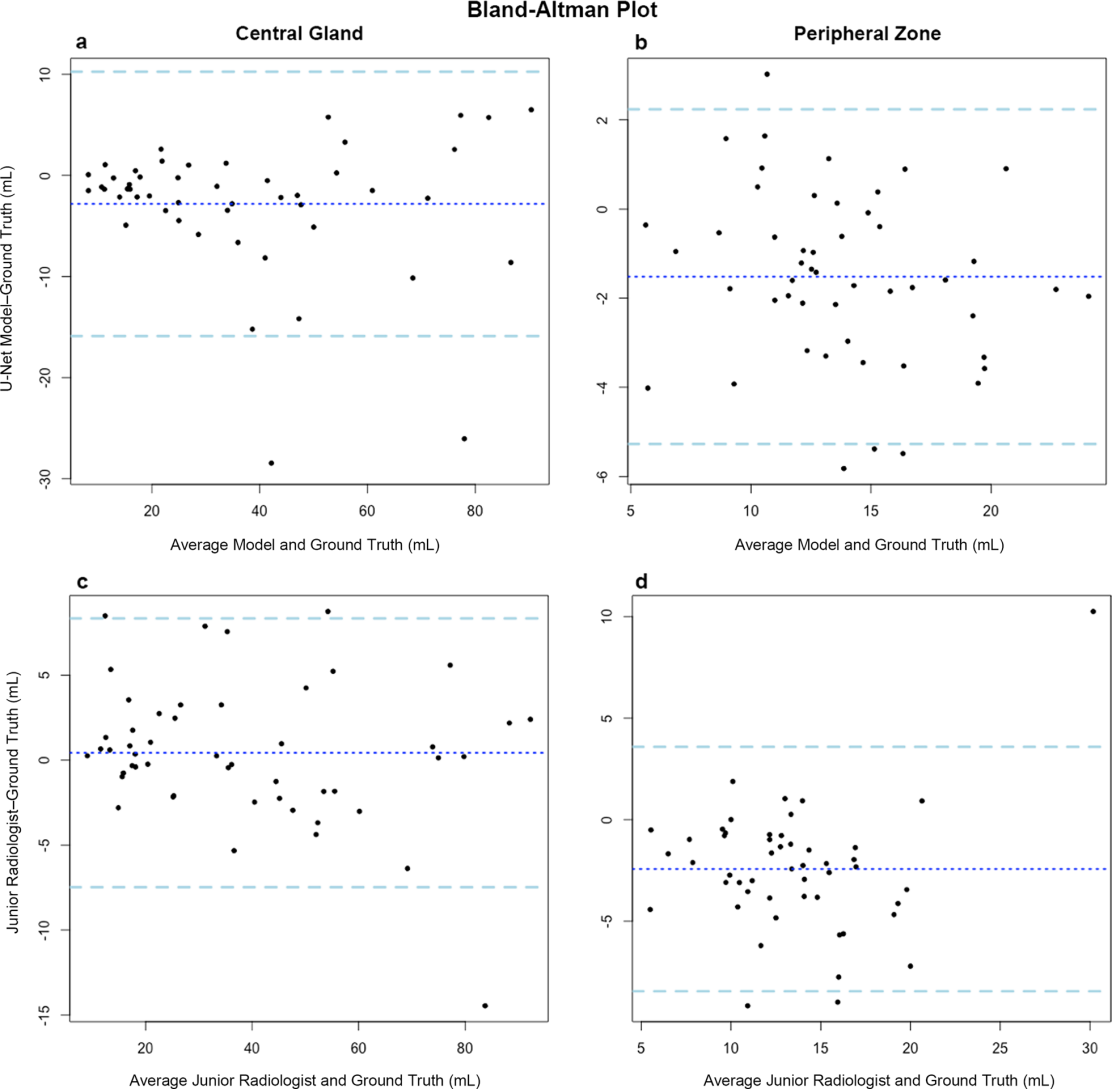
The Bland–Altman plots for the agreement between the U-Net model and ground truth (**a**, **b**), and between the junior radiologist and expert radiologist (**c**, **d**) for prostate central gland and peripheral zone volume estimation. The dashed blue lines represent the average difference. The dashed light-blue lines show the standard deviations of the difference below and above the average difference

As shown in Additional file 1: Table S2, the U-Net model showed comparable performance with the junior radiologist in CG countering (DSC: 0.883 for model, and 0.868 for junior radiologist, *p* = 0.149), but significantly better performance in PZ countering (DSC: 0.769 for model, and 0.706 for junior radiologist, *p* < 0.001). As for prostate zonal volume estimation, the U-Net model had higher agreement with the ground truth than the junior radiologist for PZ (ICC: 0.836 vs. 0.668) but showed slightly lower agreement for CG (ICC: 0.953 vs. 0.985). Bland–Altman plots (Fig. 3) showed that most volume difference was within the standard deviation of the average difference. We also observed a smaller volume difference bias of PZ between the U-Net and ground truth than between the junior radiologist and ground truth.

### Factors influencing automated zonal segmentation

**Fig. 4:**
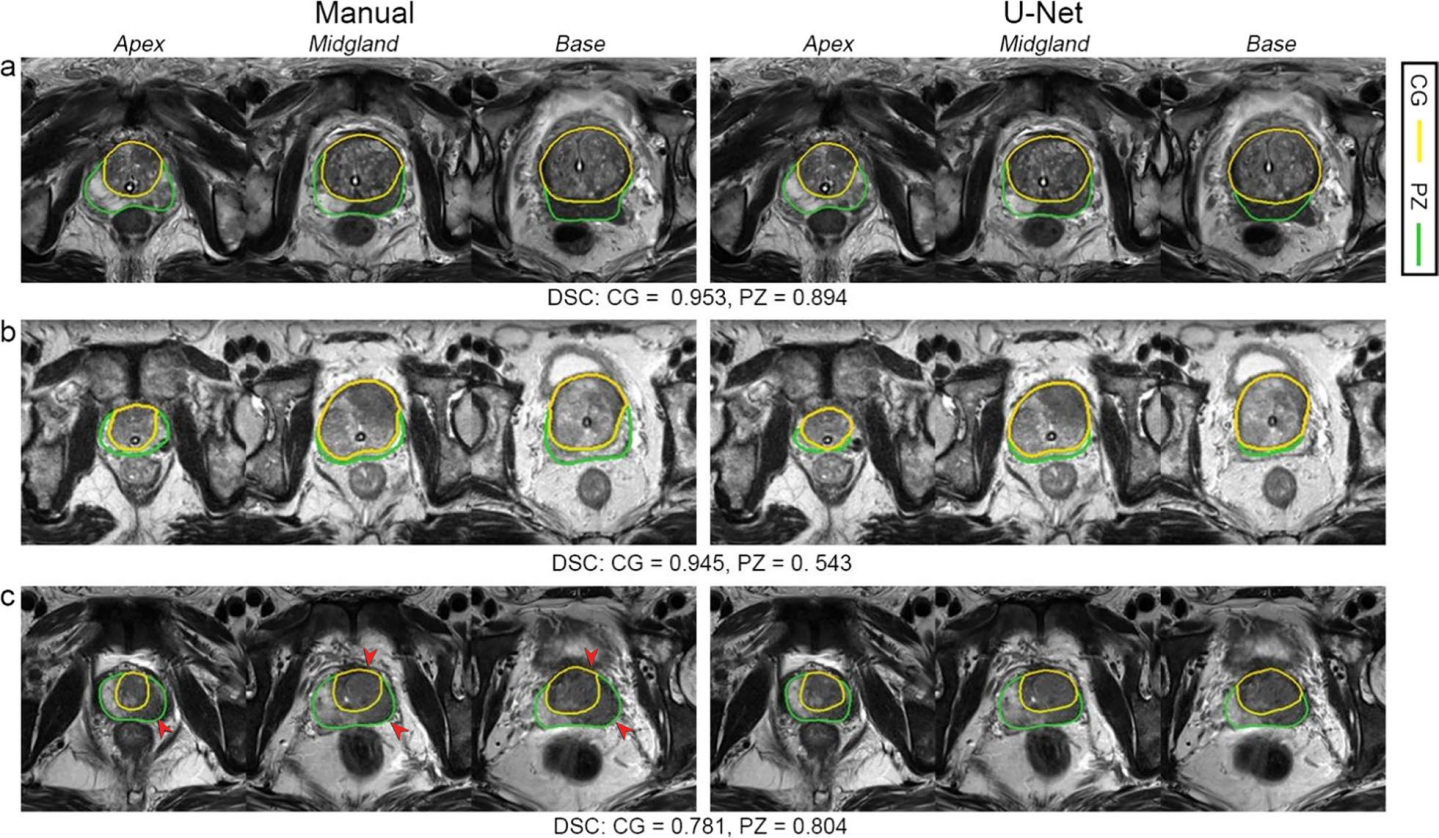
Examples of U-Net segmentation performance influenced by prostate morphology. In case **a**, the prostate is hyperplastic with increased prostate volume and the peripheral zone (PZ) is still identifiable; the U-Net model shows good segmentation for both the central gland (CG) and PZ, with Dice similarity coefficients (DSCs) of 0.953 and 0.894, respectively. In case **b**, the PZ is compressed by CG, with increased CG/PZ volume ratio; the segmentation of PZ is challenging, with a DSC of 0.543. In case **c**, despite tumor involving both PZ and CG (arrow heads) and blurring the boundary between PZ and CG, the U-Net model can generate the zonal outline, thereby offering benefit for the localization of prostate lesions

Multivariate regression analysis showed that prostate morphology and MR imaging parameters had an impact on prostate zonal segmentation. The mean DSC of CG was significantly higher in patients with larger CGv (*p* < 0.001, respectively), and was higher for images acquired from 3.0T MR scanners and the same vendor with the training group (*p* = 0.031 and 0.004, respectively) (Table 3). As for PZ segmentation, the mean DSC was significantly higher in patients with larger CGv and smaller CGv/WGv (*p* = 0.011 and < 0.001, respectively), and was higher for images acquired from the same MR vendor with the training group (*p* = 0.040) (Table 3). Figure 4 illustrates the effect of prostate morphology on the U-Net auto-delineation performance.

**Table 3:** Multivariate regression analyses of factors affecting Dice similarity coefficient in the private external testing group

	CG				PZ			
Impact factors	Estimate	Lower (2.5%)	Upper (97.5%)	*p* value	Estimate	Lower (2.5%)	Upper (97.5%)	*p* value
Tumor location	− 0.179	− 0.471	0.114	0.230	− 0.092	− 0.541	0.357	0.690
Lesion diameter	− 0.062	− 0.192	0.069	0.354	− 0.030	− 0.205	0.145	0.736
PI-RADS	− 0.063	− 0.292	0.167	0.594	− 0.048	− 0.372	0.276	0.771
T stage	− 0.087	− 0.517	0.343	0.691	0.017	− 0.617	0.651	0.958
PZv	− 0.027	− 0.053	− 0.000	0.046	− 0.032	− 0.068	0.004	0.082
CGv	0.026	0.012	0.041	** < 0.001**	0.022	0.005	0.038	**0.011**
CGv/WGv	− 1.557	− 3.709	0.596	0.156	− 5.202	− 8.060	− 2.344	** < 0.001**
Sphericity	2.192	− 3.149	7.532	0.421	− 4.066	− 11.973	3.842	0.314
Presumed circle area ratio	− 3.157	− 7.604	1.290	0.164	− 2.973	− 9.482	3.535	0.371
MR field strength	0.536	0.049	1.023	**0.031**	0.063	− 0.698	0.824	0.872
Vendor	0.524	0.171	0.878	**0.004**	0.545	0.024	1.066	**0.040**
Slice thickness	− 0.311	− 0.625	0.003	0.052	0.221	− 0.242	0.684	0.349
Pixel spacing	0.459	− 0.053	0.971	0.079	− 0.241	− 1.043	0.562	0.557

Significant *p* values (< 0.05) are shown in bold. *PZ* Peripheral zone, *CG* Central gland, *PI-RADS* Prostate Imaging-Reporting and Data System, *PZv* Peripheral zone volume, *CGv* Central gland volume, *WGv* Whole gland volume

## Discussion

Zonal segmentation is important in the management of prostatic diseases. Many studies have demonstrated the feasibility of training CNN models for zonal segmentation. However, they lack validation in non-public datasets and consideration of the patients’ characteristics. The application performance in patient cohorts with different clinicopathological characteristics remains unknown. Moreover, factors influencing the segmentation performance have rarely been investigated. In this study, we trained a 3D U-Net model for prostate zonal segmentation and applied two external testing datasets to assess its clinical utility in different patient cohorts. The model yielded good performance in all testing groups and outperformed the junior radiologist for PZ segmentation with higher DSC and ICC for volume estimation. The model performance was demonstrated to be susceptible to the prostate morphology and MR scanner parameters.

Our trained U-Net model showed good performance in zonal segmentation in both ETD_pub_ and ETD_pri_. Previously reported mean DSCs for CG and PZ segmentation in public external datasets were 0.80–0.90, and 0.64–0.81 [8, 19, 20] respectively. Our model also showed a good result in a public dataset with mean DSC of 0.889 and 0.755 for CG and PZ, respectively. In the private external testing dataset, which consisted of advanced prostate cancer, the U-Net model also showed promising results. Regardless of tumor extension, the U-Net model can recognize the natural border of prostate anatomy zones with high consistency with radiologist (Fig. 4), which could serve as the foundation for orientation of prostate tumor and identification of extraprostatic cancer. Compared with previous studies testing CNN model’s performance in private external testing datasets [21, 22], our study applied the model to patients with different clinic scenarios and considered the patients’ clinicopathological characteristics. Furthermore, even without the fine-tuning process [21], our trained model still showed good performance in external testing. Our study also showed that segmentation in the extreme parts of prostate is challenging. Specifically, among different testing groups, the mean DSCs in the apex, base, and midgland of the prostate were 0.811–0.856, 0.847–0.901, and 0.916–0.941 for CG, and were 0.625–0.788, 0.739–0.832, and 0.818–0.896 for PZ, respectively. Other studies have also reported a significantly lower DSC in the apex and base of the prostate even for radiologists’ manual delineation, with DSC of 0.85 in the apex, 0.87 in the basal part, and 0.89 in the midgland [23].

The U-Net model outperformed the junior radiologist in PZ segmentation with a significantly higher DSC and better agreement of volume estimation, and was comparable to the junior radiologist in CG segmentation. In our study, the volume estimation ICCs of the U-Net model and the expert radiologist’s manual segmentation were 0.836 for PZ and 0.953 for CG, which were close to the literature-reported values of a radiologist’s manual segmentation between two MR scans in one patient cohort (0.888 for PZ and 0.988 for non-PZ) [24]. The volume calculation variability was higher in PZ than in CG, due to the irregular morphology of PZ. The ICC for CG volume estimation of the junior radiologist in our study was excellent, while the ICC for PZ volume estimation showed moderate agreement. The junior radiologist lacked a good grasp of the prostate anatomy and included some periprostatic fat as PZ, which led to the overcalculation of PZ volume. The prostate volume estimation is an important biomarker for multiple clinical applications [25, 26]. Lee et al. suggested that volume measurement by automated network provided reliable volume estimates of the prostate compared with those obtained with the ellipsoid formula [10]. Our study demonstrated that our automated network was able to provide faster and more accurate prostate zonal volume calculation than the junior radiologist, especially in PZ, which could serve as a useful tool for accurate prostate-specific antigen density calculation and obstructive symptoms analysis of patients.

Prostate morphology affected the segmentation performance of the U-Net model. In our study, the DSC for both CG and PZ was higher for larger CGv, while the DSC for PZ was lower for larger CGv/WGv. Prostate hyperplasia is common in men and is age-related, which contributes to the increase in CGv and the compression of PZ. Therefore, the recognition of CG was easier, but with extreme compression of PZ, the segmentation of PZ became more difficult. Prostate morphology also has an influence on manual segmentation variance. Montagne et al. [7] reported the variability of manual prostate zonal segmentation by seven radiologists on T2WI, with DSC value of 0.88–0.91 for TZ and analyzed factors that may influence it. The results showed that the DSC was lower for smaller prostate (Spearman correlation ρ > 0.8). Nai et al.[12] found that CNN auto-segmentation was difficult for special cases, which was the most difficult for cases with transurethral resection of the prostate. However, since the number of special cases was small in their study (only four subjects), no statistical analysis was provided. Rouvière et al.’s [17] study found a discordant result, where the mean DSC value for CG segmentation decreased significantly when the CG volume increased. The decreased performance of their model for larger prostate might be due to the different training process, combining model-based and deep learning–based approaches.

MR imaging parameters also significantly influenced the model’s auto-segmentation performance. The DSC value for PZ and CG was significantly higher in images acquired from the same vendor with the training group. Furthermore, the DSC value for CG was significantly higher for images from 3.0T MR scanners. In a previous study, Rouvière et al. [17] found that the scanner used for imaging significantly influenced the mean DSC for CG segmentation, with an odds ratio of 0.69 (1.5T vs. 3.0T). Since the MR scanners affect the model’s auto-segmentation, further training of the model with heterogeneous datasets might be necessary. In our study, the patients’ clinicopathological information was less likely to affect the segmentation performance. The reasons may be the relatively homogeneous clinicopathological data in the ETD_pri_, since all patients were diagnosed with advanced prostate cancer. Further studies using a larger cohort with heterogeneous patient data might be necessary. Additionally, a previous study has reported the change in prostate morphology by using the endorectal coil [27]. However, none of the patients included in our study used an endorectal coil. Whether our model is applicable to these patients should be analyzed in future studies.

Our study has some limitations. First, the private external testing dataset was small, so further external testing using larger datasets is needed. Furthermore, the other structures of the prostate, such as the anterior fibromuscular stroma and seminal vesicles, were not segmented since their outlining is difficult. However, for more accurate prostate cancer staging, the segmentation of seminal vesicles should be considered in future studies. Finally, the manual segmentation to generate the ground truth is time-consuming, which also limited the cases used for analysis; thus, utilizing the model to generate the ground truth in future studies is worth trying.

In conclusion, we validated the model’s utility for prostate zonal segmentation on T2WI in different external testing datasets. The model yielded good performance regardless of the variations in the patients’ clinicopathological characteristics. The model showed better performance than the junior radiologist in PZ segmentation. Prostate morphology and MR scanner parameters, especially CGv and vendor, impact zonal segmentation performance.

## Supplementary Information

**Additional file 1:** Ground truth segmentation, prostate zonal segmentation model, and supplementary tables.

## Data Availability

The datasets used and/or analyzed during the current study are available from the corresponding author on reasonable request.

## Abbreviations

95HD: 95th Hausdorff distance
ABD: Average boundary distance
CG: Central gland
CGv: Central gland volume
CNN: Convolutional neural networks
DSC: Dice similarity coefficient
ETD_pri_: Private external testing dataset
ETD_pub_: Public external testing dataset
ICC: Intraclass correlation coefficient
PZ: Peripheral zone
PZv: Peripheral zone volume
WGv: Whole gland volume
