# Supplementary material for: Development and clinical utility analysis of a prostate zonal segmentation model on T2-weighted imaging: a multicenter study
Source: Insights Imaging. 2023 Mar 16;14:44. doi: 10.1186/s13244-023-01394-w (PMC10020392; doi:10.1186/s13244-023-01394-w)
Supplement: Supplementary file 1 — Additional file 1: Ground truth segmentation, prostate zonal segmentation model, and supplementary tables. [file 13244_2023_1394_MOESM1_ESM.pdf]

ELECTRONIC SUPPLEMENTARY MATERIAL

Development and clinical utility analysis of a prostate zonal segmentation model on T2-weighted imaging: a multicenter study

S-1. Ground truth segmentation

One expert radiologist manually segmented the central gland (CG) and peripheral zone (PZ) to serve as the ground truth. The CG included TZ and central zone and anterior fibromuscular stroma. The PZ was obtained by subtracting the whole gland and the CG. For lesions involving the CG and PZ simultaneously, or expanding the border of prostate, the radiologist delineated the natural margin of the prostate, therefore, the delineation could serve as the foundation of lesion orientation and extraprostatic lesion identification.

S-2. Prostate zonal segmentation model

Pre-processing

The images were resampled to the median pixel spacing of the training cohort (3.00 × 0.51 × 0.51) to offset the bias caused by resolution inconsistency, and then they were cropped into the input patch (14 × 352 × 352, determined by the average non-zero area of images) for the segmentation model. Finally, the intensity of the images was normalized by z-score normalization.

Convolutional neural network

3D U-Net-based prostate zonal segmentation model was trained by self-configuring nnU-Net framework. For each pixel of the input image, the model predicted three probabilities for the non-prostatic region, the CG region, and the PZ region, respectively. Then the label corresponding to the maximum probability was assigned to this pixel.

In the training stage, the online data augmentation methods including mirroring, scaling, rotation, and translation were applied to avoid over-fitting. With an epoch number of 500, an initial learning rate of 0.01, and a batch size of 2, the prostate zonal segmentation model was trained in 5-fold cross-validation procedure. The loss function was the combination of the Dice loss and the Binary Cross Entropy loss. Then in the inference stage, the ensemble results of the 5 models trained in the 5-fold cross-validation procedure were used as the mode prediction.

Table S1. MRI acquisition parameters for axial T2-weighted imaging (median [range]).

Dataset	n	Vendor	Scanner	MR field strength (T)	TR (ms)	TE (ms)	Matrix	Slice thickness (mm)	Pixel spacing (mm^2)
Training group	223	GE	DISCOVERY 750	3.0	4422 (2672–5367)	108 (97–116)	512 × 512	3 (3–6)	0.51 (0.43–0.78)
Internal testing group	93	GE	DISCOVERY 750	3.0	4424 (4117–5534)	108 (86–109)	512 × 512	3 (3–4)	0.51 (0.51–0.53)
ETD_{pub}	141	Siemens	Skyra TrioTim	3.0	5660 (4480–8624)	104 (101–104)	256 × 256–640 × 640	3 (3–5)	0.50 (0.3–0.7)
ETD_{pri}	59	Siemens Philips GE	Espree/Skyra/Aera; Achieva; DISCOVERY 750/Optima 360/ EXCITE/Pioneer	1.5/3.0	5183 (2500–7970)	104 (80–122)	256 × 256–672 × 672	3.5 (3–5)	0.47 (0.30–0.78)

Note—ETD_{pub} = public external testing dataset, ETD_{pri} = private external testing dataset, TR = repetition time, TE = echo time.

Table S2. Dice similarity coefficient, 95th Hausdorff distance, average boundary distance and volume variability of 3D U-Net model and the junior radiologist on T2-weighted images in fifty randomly selected patients from ETD_{pub}.

	CG				PZ			
	DSC	95HD (mm)	ABD (mm)	Volume variability (ICC)	DSC	95HD (mm)	ABD (mm)	Volume variability (ICC)
Junior radiologist vs. GT	0.868 ± 0.061	4.432 ± 1.612	1.064 ± 0.426	0.985	0.706 ± 0.103	7.923 ± 5.179	1.261 ± 0.668	0.668
U-Net vs. GT	0.883 ± 0.052	4.654 ± 3.164	1.008 ± 0.667	0.953	0.769 ± 0.085	4.888 ± 3.369	0.879 ± 0.585	0.836
p value	0.149	0.738	0.554	-	< 0.001	< 0.001	< 0.001	-

Note—ETD_{pub} = public external testing dataset, CG = central gland, PZ = peripheral zone, DSC = Dice similarity coefficient, 95HD = 95th Hausdorff distance, ABD = average boundary distance, ICC = intraclass correlation coefficient, GT = ground truth.

The *p* values were calculated by paired *t*-test.
